# Association between anorexia and hyposalivation in community-dwelling older adults in Japan: a 6-year longitudinal study

**DOI:** 10.1186/s12877-020-01905-0

**Published:** 2020-11-25

**Authors:** Yuki Ohara, Hisashi Kawai, Maki Shirobe, Keiko Motokawa, Yoshinori Fujiwara, Hunkyung Kim, Kazushige Ihara, Shuichi Obuchi, Ayako Edahiro, Masanori Iwasaki, Yutaka Watanabe, Hirohiko Hirano

**Affiliations:** 1 Research Team for Promoting Independence and Mental Health, Tokyo Metropolitan Institute of Gerontology, 35-2 Sakae-cho, Itabashi-Ku, Tokyo, 173-0015 Japan; 2 Research Team for Human Care, Tokyo Metropolitan Institute of Gerontology, Tokyo, Japan; 3 The Tokyo Metropolitan Support Center for Preventative Long-term and Frail Elderly Care, Tokyo Metropolitan Institute of Gerontology, Tokyo, Japan; 4 Research Team for Social Participation and Community Health, Tokyo Metropolitan Institute of Gerontology, Tokyo, Japan; 5 Department of Social Medicine, Hirosaki University Graduate School of Medicine, Aomori, Japan; 6 Department of Oral Health Science, Gerodontology, Faculty of Dental Medicine, Hokkaido University, Sapporo, Japan

**Keywords:** Anorexia, Oral manifestations, Appetite, Salivation, Aged

## Abstract

**Background:**

Hyposalivation is associated with the nutritional status. Anorexia of ageing, defined as an age-related decrease in appetite and food intake, presents even in healthy adults and is considered an independent predictor of malnutrition, frailty, and mortality. However, the relationship between anorexia and hyposalivation of ageing is unclear. Thus, the present longitudinal study aimed to investigate the incidence of hyposalivation and its relationship with anorexia in community-dwelling older people in Japan.

**Methods:**

The study population comprised 220 individuals (80 men and 140 women) aged 65–86 years at baseline. The participants underwent comprehensive health check-ups, including dental examinations and anthropometry, and face-to-face interviews in 2013 and 2019. Hyposalivation was determined on the basis of the unstimulated salivary flow rate measured using the modified cotton roll method. Anorexia was defined as a score of ≤29 in the Japanese version of the Council on Nutrition Appetite Questionnaire. Logistic regression analyses were used to test whether the presence of anorexia at baseline was an independent predictor of hyposalivation.

**Results:**

Hyposalivation developed at a rate of 19.5% during the 6-year observation period. Anorexia was observed in 95 (43.2%) participants at baseline. After adjusting for potential confounding factors, anorexia (adjusted odds ratio [AOR], 2.65; 95% confidence interval [CI], 1.26–5.57) and polypharmacy (AOR, 3.29; CI, 1.06–10.19) were significant predictors of hyposalivation.

**Conclusion:**

Loss of appetite is independently correlated with and a risk factor for hyposalivation in older adults. Anorexia of ageing may have negative effects on the salivary flow rate in such settings. Salivation should be a standard feature in clinical assessments of the older adults.

## Background

Saliva plays an important role in the regulation of oral health because it helps in the maintenance of a neutral oral pH and is a reservoir of calcium and phosphate ions that are required for tooth remineralization [1]. Hyposalivation, an objective, measurable decrease in the flow of saliva, is highly prevalent in older adults [2]. In a previous study, hyposalivation was reported as a risk factor for dental caries and periodontal disease [3]. Furthermore, saliva is essential for adequate functioning of the body as it helps soften food, forms a bolus for chewing and swallowing, facilitates speech, cleans the oral tissues, and protects against tooth damage [1, 4]. Therefore, screening and early management of hyposalivation is vital for ensuring oral health, especially in individuals living in a rapidly ageing society.

Saliva contains digestive enzymes and allows for perception of the taste of foods and other substances [4]; therefore, it is also strongly associated with nutritional intake. In previous Japanese studies, older people with hyposalivation showed lower intake of certain foods, including vegetables and seafood. Hyposalivation has been associated with problems of taste perception, willingness to eat and enjoyment of meals, quality of life, and malnutrition [3, 5–7].

Anorexia of ageing is defined as an age-related decrease in appetite and food intake, and its manifestations overlap with hyposalivation. While anorexia of ageing can present in healthy older people, it is also associated with undernutrition, frailty, and mortality and, thus, can be a key indicator of the nutritional status [8]. Although Kimura et al. recently reported a significant association between anorexia and masticatory function [9], its association with hyposalivation remains unclear. Therefore, the aim of this longitudinal study was to investigate the incidence of hyposalivation and its relationship with anorexia in community-dwelling older people in Japan.

## Methods

### Study population

**Fig. 1:**
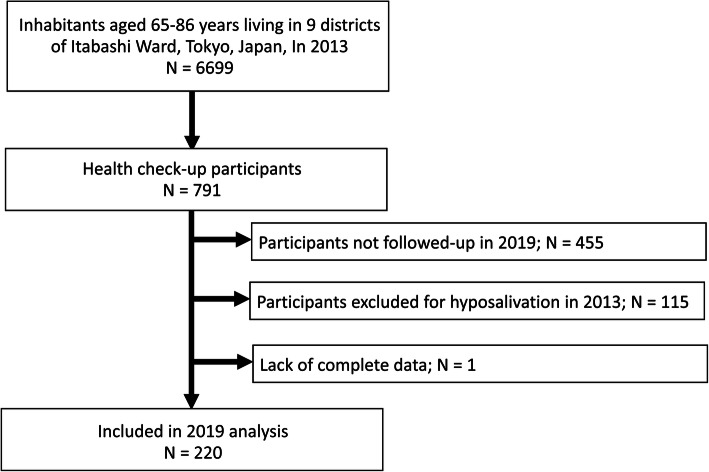
Flow chart of the participant recruitment process

Details on the participant recruitment process are presented in Fig. 1. Longitudinal data were derived from the Otassha-Kenshin, a community-based cohort study conducted by the Tokyo Metropolitan Institute of Gerontology. The design and logistics of the study have been described in detail elsewhere [10, 11]. The participants underwent a comprehensive health check-up in 2013, which was repeated 6 years later. The length of the follow-up period was determined from results of previous studies focusing on hyposalivation among community-dwelling older people [4, 12]. The original 2013 cohort comprised 791 individuals (340 men and 451 women; mean age: 73.5 [standard deviation, 5.6] years). Individuals with hyposalivation at baseline (in 2013), those who did not participate in the follow-up survey, and those with missing data regarding the salivary flow rate in 2019 were excluded. The final study comprised 220 individuals (80 men and 140 women). This study was approved by the ethics committees of the Tokyo Metropolitan Institute of Gerontology (approval Nos. H23–1253 and R1-JIN15). Written informed consent was obtained from all participants and the study was performed in accordance with the Declaration of Helsinki.

### Assessment of the salivary flow rate

For the determination of hyposalivation in the 2019 follow-up survey, unstimulated saliva was collected between 9:00 and 16:00 using the modified cotton roll method [13]. Participants had been instructed not to eat within 1 h of the assessment or drink water or other liquids within 30 min of the assessment. A preweighed cotton roll was placed under the tongue. The participants were instructed to close their mouth for 30 s, and the cotton roll was subsequently removed. The amount of saliva absorbed by the cotton roll was then measured using a standardized electronic scale after calibration with copper [13, 14]. The measurement was performed once for each participant. Hyposalivation was defined as an unstimulated saliva level of < 0.1 g, in accordance with previous studies [13, 15].

### Assessment of the oral health status

All oral health assessments were performed by two dentists and 10 dental hygienists who had attended a 2-h training session. The training manual included an overview of the study and appropriate examination and data collection methods, and it had been constructed by the authors for the survey. For assessment of the interexaminer agreement, standardized evaluation criteria were developed by the first author, and the examiners were trained using volunteer participants until agreement with the criteria was achieved.

All assessments were conducted in a multipurpose room within our institute, with participants sitting on a chair with their head upright. The number of teeth present was recorded under appropriate illumination using artificial light. As severely decayed teeth, teeth with pulp decay, and tooth stumps are not useful for mastication, they were excluded from the count [16]. Occlusal force was measured by placing a horseshoe-shaped pressure-sensitive film (Dental Prescale 50H®) between the teeth, and participants were instructed to bite it with maximum force. Subsequently, the film was analyzed with a computerized measurement system (Dental Prescale Occluzer®) that determined the maximum occlusal force [17]. The repetitive saliva swallowing test (RSST), which assesses swallowing function, was administered and involved measurement of the frequency with which patients swallowed their saliva over a 30-s period [18]. The presence of xerostomia—the subjective feeling of oral dryness—was assessed with the dichotomous question “Does your mouth feel dry?”, based on the Kihon Checklist prepared by the Japanese Ministry of Health and Welfare [15, 19].

### Appetite questionnaire

The participants’ appetite was measured in both 2013 and 2019 using a validated Japanese version of the Council on Nutrition Appetite Questionnaire (CNAQ-J) [20]. The CNAQ-J comprises eight questions on the following items: appetite, feeling full, feeling hungry, food tastes, food tastes compared to those experienced when younger, meal frequency per day, feeling sick or nauseated when eating, and usual mood. The participants were requested to respond to each question on a 5-point Likert scale (1–5). The total score in the CNAQ-J ranges from 8 to 40 points, with lower scores indicating appetite deterioration. The reliability and validity of the CNAQ-J have been established previously [20]. Anorexia was defined as a CNAQ-J score of ≤29 [21].

### Covariates

We considered factors previously reported to be associated with salivary secretion and anorexia as covariates [1–9, 12, 14, 15]. Information on the participants’ age, sex, smoking status, and frequency of going out was collected in 2013 using a self-administered questionnaire. The frequency of going out, included as an evaluation of the participants’ level of physical activity, was assessed with the dichotomous question “Do you go out at least once a week?” [19]. In addition, trained nurses interviewed the participants regarding comorbidities and the intake of prescription medications. The use of five or more prescribed medications was defined as polypharmacy. Depressive state was evaluated by the self-administered Zung Self-Rating Depression Scale (SDS) [22]. Body composition was evaluated using the body mass index (BMI), calculated as the body weight divided by the height squared. Blood samples were drawn for the measurement of serum albumin levels.

### Statistical analyses

Statistical analyses were performed using IBM SPSS Statistics, version 25.0. The level of significance was set at α = 0.05. Participants were classified according to the presence of hyposalivation at the 6-year follow-up, and their characteristics were compared with those observed at baseline. Continuous variables were tested for normality, and Student’s t-test or the Mann–Whitney U test was used for between-group comparisons. Categorical variables were compared using chi-squared or Fisher’s exact tests, as appropriate. The Wilcoxon signed-rank test was used to compare changes in CNAQ-J scores between the baseline and follow-up periods. For examination of the association between anorexia and hyposalivation at baseline, logistic regression analysis with forced covariate entry was performed. Baseline variables were included as covariates if they had a *p*-value of < 0.25 in bivariate analyses. Although age and sex had nonsignificant *p*-values in the bivariate analyses, they were forced into the models. To avoid the effects of multicollinearity, we confirmed that none of the potential covariates had an intercorrelation coefficient of > 0.8.

## Results

Table 1 summarizes the participants’ baseline characteristics, grouped by their hyposalivation status in 2019. Over the 6-year follow-up period, 19.5% participants developed hyposalivation; no significant differences were observed with respect to sex or age. The incidence of anorexia at baseline in the hyposalivation group was 60.5%, which was significantly higher than that observed in the non-hyposalivation group (39.0%; *p* = 0.001). No significant differences were observed between the groups in baseline serum albumin or BMI levels. Similarly, in terms of oral health status at baseline, there were no significant differences between the groups in the number of teeth present, occlusal force, or swallowing function. The prevalence of xerostomia was 23.1%; the difference between the groups was not significant.

**Table 1:** Baseline characteristics of the participants

	Total *N* = 220		No Hyposalivation *N* = 177 (80.5%)		Hyposalivation *N* = 43 (19.5%)		*p*-value^a^
Demographic and general health status							
Age, years, median (IQR)	72	(69–76)	72	(69–76)	72	(69–76)	.941^b^
Sex, female, n (%)	140	(63.6)	111	(62.7)	29	(67.4)	.563^c^
Body mass index, kg/m^2^, median (IQR)	22.5	(20.8–24.9)	22.4	(20.8–24.8)	22.6	(20.9–25.6)	.552^b^
Anorexia, n (%)	95	(43.2)	**69**	**(39.0)**	**26**	**(60.5)**	**.001**^**c**^
Serum albumin, mg/dL, median (IQR)	4.3	(4.2–4.4)	4.3	(4.2–4.4)	4.3	(4.2–4.5)	.556^b^
SDS, point, median (IQR)	28	(24–32)	27	(24–31)	30	(24–35)	.101^b^
Current smoker, n (%)	23	(10.5)	18	(10.2)	5	(11.6)	.779^d^
Number of comorbidities, median (IQR)	1	(0–2)	1	(0–2)	1	(0–2)	.937^b^
Polypharmacy, n (%)	19	(8.6)	12	(6.8)	7	(16.3)	.066^c^
Going out at least once a week, n (%)	217	(98.6)	175	(98.9)	41	(95.3)	.121^d^
Oral health status							
Number of present teeth, median (IQR)	24	(18–27)	24	(18–27)	25	(19–27)	.882^b^
Occlusal force, N, median (IQR)	404.0	(223.2–568.3)	404.2	(231.9–570.9)	386.8	(150.6–560.6)	.350^b^
RSST (times/30 s), median (IQR)	4.0	(3.0–5.0)	4.0	(3.0–5.0)	4.0	(3.0–5.0)	.343^b^
Xerostomia, n (%)	51	(23.2)	38	(21.5)	12	(27.9)	.366^c^

Values are median (interquartile range) for continuous variables or n (%) for categorial variables. SDS, Zung Self-Rating Depression Scale; RSST, repetitive saliva swallowing test; IQR, interquartile range

Bold text indicates statistically significant associations (*p* < 0.05)

^a^ *p*-value for the comparison between groups

^b^ Mann–Whitney U test

^c^ chi-square test

^d^ Fisher’s exact test

Table 2 shows the changes in participants’ appetite over the 6-year follow-up period. Neither group showed significant appetite-related changes as measured by the CNAQ-J scores over the 6-year observational period.

**Table 2:** Changes in appetite during the 6-year follow-up and comparison between the non-hyposalivation and hyposalivation groups

	Non-hyposalivation N = 177	*p*-value^a^	Hyposalivation N = 43	*p*-value^a^	*p*-value^b^
CNAQ-J, points, median (IQR)					
Baseline	30 (29–32)	0.615^c^	29 (28–30)	0.104^c^	0.007^d^
Follow-up	30 (28–32)		29 (27–30)		0.001^d^

CNAQ-J, Japanese version of the Council on Nutrition Appetite Questionnaire; IQR, interquartile range

^a^ *p*-value for the comparison between baseline and follow-up period

^b^ *p*-value for the comparison between the non-hyposalivation and hyposalivation groups

^c^ Wilcoxon signed-rank test

^d^ Mann–Whitney U test

Table 3 summarizes the results of multiple logistic regression analysis of the relationship between anorexia and hyposalivation. After adjusting for potential confounding factors, anorexia was a significant predictor of hyposalivation (adjusted odds ratio [AOR], 2.65; 95% confidence interval [CI], 1.26–5.57), as was polypharmacy (AOR, 3.29; CI, 1.06–10.19).

**Table 3:** Unadjusted and adjusted logistic regression models for related covariates and hyposalivation

	Unadjusted logistic regression			Adjusted logistic regression		
	OR	95% CI	*p*-value	Adjusted OR	95% CI	*p*-value
Anorexia (for presence)	2.39	1.21–4.73	0.012	2.65	1.26–5.57	0.010
Age, one increment	0.99	0.92–1.06	0.712	0.95	0.88–1.02	0.184
Sex (male = 0, female = 1)	1.23	0.61–2.50	0.563	1.08	0.51–2.29	0.834
SDS, one increment	1.04	0.99–1.08	0.093	1.01	0.96–1.06	0.803
Polypharmacy (for presence)	2.67	0.98–7.26	0.054	3.29	1.06–10.19	0.039
Going out at least one a week (for yes)	4.27	0.58–31.20	0.153	5.05	0.62–40.78	0.129

Forced entry analysis. *OR* odds ratio, *CI* confidence interval, *SDS* Zung Self-Rating Depression Scale

## Discussion

To our knowledge, this longitudinal study is the first to examine the incidence of hyposalivation using unstimulated salivary flow measurements, and to report the association between anorexia and hyposalivation in older community-dwelling people. Several longitudinal studies have investigated the factors associated with “dry mouth” based on the presence of xerostomia, which is a subjective complaint related to salivary flow and for which data collection is simpler than for hyposalivation [23–26]. Consistent with previous studies, we did not find a significant association between hyposalivation and xerostomia [2, 15, 27], implying that they are measuring different aspects of salivary secretion. Although hyposalivation appears to be a more rigorous assessment of age-related changes in salivary secretion, few longitudinal studies have investigated its incidence in older community-dwelling adults [2, 12].

In the present study, hyposalivation developed in nearly 20% of a relatively healthy older persons. Since saliva not only preserves the health status of the oral cavity but is also involved in the maintenance of general health status [3, 5, 7, 28], these findings highlight the importance of early screening and management in older adults. Both xerostomia and hyposalivation are considered to have negative impacts on the oral cavity [3, 29], and the assessment of both conditions is important in clinical settings. Our assessment of xerostomia was based on a dichotomous question; future studies should consider the use of Likert-like scales for a more precise assessment of the degree of dryness.

The findings of the present study suggest that anorexia of ageing influenced the incidence of hyposalivation even after adjustment for age, sex, medication, and psychological status [3, 5, 12, 15]. Neither the hyposalivation group nor the non-hyposalivation group showed significant appetite-related changes, as measured by the CNAQ-J scores, over the 6-year observational period. However, a relationship between the two was observed in the longitudinal analyses. Thus, these results support an independent effect of baseline anorexia on the development of hyposalivation.

The observed association between hyposalivation and medication intake was consistent with the findings of previous studies [15, 30, 31]. Previous studies have reported that both anorexia of ageing and hyposalivation are associated with psychological factors [2, 30, 32]. However, in our study, decreased appetite was an independent risk factor for hyposalivation during the 6-year period, even after controlling for the effects of depressive symptoms. Anorexia of ageing is a factor that contributes to undernutrition and adverse health outcomes; therefore, interventions aimed at improving appetite should be implemented.

The primary triggers of appetite are smell and taste, and the latter greatly affects the rate of salivary secretion [8]. Salivary function is induced by mastication and gustatory stimuli [29]. The taste pathway is activated by impulses from the facial, glossopharyngeal, and vagal nerves, which have ipsilateral connections to the salivatory centers in the brainstem [29].

Generally, there is a difference between the unstimulated saliva secreted on autonomic stimulation and stimulated saliva secreted during chewing. Unstimulated saliva contains several tasting compounds, and these compounds may constantly stimulate the taste receptors located on the tongue [33]. One hypothesis that may explain our results is that the decrease in appetite induced by taste perceptions affects the amount of unstimulated saliva, and that measures aimed at improving patients’ drive to eat and enjoyment of meals may lead to an increased salivary flow rate.

The major salivary glands secrete saliva for the lubrication and protection of the oral cavity in response to mucosal dryness as well as low-grade mechanical stimulation associated with lip and tongue movements [31]. Wang et al. reported that frequent gum-chewing is associated with unstimulated salivary flow [34]; thus, habitual oral movements may promote not only the rate of stimulated saliva secretion but also that of unstimulated saliva secretion.

Decreased appetite, starting with an inability to perceive taste, and reduced daily oral movements are associated with hyposalivation [4, 29, 34]. The mechanism underlying the association between anorexia and hyposalivation was not evaluated in detail in this study because appetite was evaluated using a self-administered questionnaire. Validation with other investigative modalities, such as gustometry, is necessary in the future. Previous studies have reported that the nutritional status may also affect salivation [5, 6]; however, there were no significant associations with nutritional indices such as BMI and serum albumin level in the present study. Because this study included relatively healthy community-dwelling older adults, a relatively high BMI and serum albumin level would have been common. Further investigations using specific nutritional assessment indicators such as nutritional intake are warranted.

The limitations of this study should be recognized for accurate interpretation of the results. First, the sample comprised independently living individuals who volunteered to participate in the health examination; therefore, they likely represented a healthier portion of the general older adults. Second, over half the original participants were lost to follow-up over the 6-year period. Although this may be attributed to several factors, such as the requirement for long-term care and relocation, it has not been analyzed in detail. Third, there were inconsistencies in the definition of hyposalivation among studies. We measured the participants’ unstimulated salivary flow rates using the cotton roll method in order to enable noninvasive data collection within a short time period [14]. In the future, it is advisable to evaluate both stimulated and unstimulated salivary flow rates in the same participants, and to develop a reliable comparison scale that can be applied clinically and in research. Finally, although salivary flow rates are known to vary during the day [35, 36], we were unable to unify the actual time of saliva assessments for each participant during the baseline and follow-up evaluations.

In conclusion, we detected the development of hyposalivation in 19.5% individuals in a cohort of older adults over a 6-year period. Further, we found that anorexia was an independent risk factor for hyposalivation. To our knowledge, this study is the first to show an effect of decreased appetite on hyposalivation. These results can be used for the development of screening or treatment protocols aimed at reducing the incidence of nutrition-related frailty in older adults.

## Data Availability

The data that support the findings of this study are available from Otassha-Kenshin Study, but restrictions apply to the availability of these data, which were used under license for the current study and, therefore, are not publicly available.

## Abbreviations

OR: Odds ratio
CI: Confidence interval
RSST: Repetitive saliva swallowing test
CNAQ-J: Japanese Version of the Council on Nutrition Appetite Questionnaire
SDS: Zung Self-Rating Depression Scale
